# Study on the association between visceral adiposity index and diabetic kidney disease in hospitalized patients with type 2 diabetes mellitus in China

**DOI:** 10.3389/fendo.2025.1549954

**Published:** 2025-03-14

**Authors:** Xin Zhao, Jianbin Sun, Sixu Xin, Xiaomei Zhang

**Affiliations:** Department of Endocrinology, Peking University International Hospital, Beijing, China

**Keywords:** type 2 diabetes mellitus, lipid accumulation product, visceral adiposity index, abdominal volume index, body round index, conicity index, diabetic kidney disease

## Abstract

**Objective:**

This study aims to explore the correlation between visceral adiposity index (VAI) and diabetes kidney disease (DKD) in patients with type 2 diabetes mellitus (T2DM), so as to provide a clinical basis for the prevention and treatment of DKD.

**Methods:**

This study retrospectively analyzed 1817 patients with T2DM hospitalized in the department of Endocrinology, Peking University International Hospital from January 2017 to August 2021, including 1053 males and 764 females. According the level of VAI, subjects were divided into three groups.

**Results:**

(1) The results showed that with the increase of VAI level, the proportion of DKD gradually increased, and there was a statistical difference (p < 0.05). With the increase of VAI levels, there is an increasing trend in males, age, WC, BMI, WHtR, WHR, VAI, LAP, ABSI, C-Index, CUN-BAE, SBP, DBP, HbA1c, FBG, PBG, UACR, TG, while HDL-C levels show a decreasing trend (p all <0.05). (2)Logistic regression showed that after adjusting age, sex, diabetic duration, smoking, drinking, BP, blood glucose and blood lipids, high level of VAI was an independent risk factor for DKD (HR=1.38, 95% CI 1.18, 1.63). (3)The model to predict the risk of DKD using anthropometric indicators, showed that the AUC of the models ranked VAI>ABSI>C-index>WHR>AVI=BRI>BMI>CUN-BAE>LAP>WHtR.(4)The predictive ability for DKD of Model 1 with VAI was higher than that of Model 2 with BMI.

**Conclusion:**

The increase of VAI is an independent predictor of DKD occurrence in patients with T2DM, which provides a certain clinical basis for preventing the development of DKD in patients with T2DM.

## Introduction

The prevalence of diabetes mellitus (DM) has been gradually increasing in recent years (1). In China, the prevalence of DM in adults increased from 10.9% in 2013 to 12.4% in 2018, with the diabetic population reaching 140 million in 2021 (2). End-stage renal disease (ESRD) is a common cause of end-stage diabetic kidney disease (DKD), imposing a significant economic burden on patients and greatly affecting long-term quality of life (3). Abundant clinical studies have confirmed that obesity, especially abdominal obesity and lipid metabolism disorders, are major risk factors influencing the development of type 2 diabetes mellitus (T2DM) and are crucial links between insulin resistance and T2DM. Therefore, finding suitable indicators to assess abdominal obesity and clues related to DKD is of significance for the management and clinical treatment of DKD in diabetic patients.

The current standard practice in clinical settings involves the widespread use of body mass index (BMI) to assess obesity levels. However, BMI, as an indicator of obesity, fails to account for variations in fat distribution. Some individuals classified as obese based on BMI may have normal metabolic profiles, while others with normal BMI may experience disruptions in glucose metabolism. To address these limitations, clinical and epidemiological studies primarily rely on measurements such as waist circumference (WC) and waist-to-hip ratio (WHR) to evaluate abdominal fat deposition. However, WC cannot differentiate between visceral and subcutaneous adipose tissues and does not adjust for height differences (4). As a result, there is ongoing development of alternative anthropometric indicators.

Visceral Adiposity index (VAI), which is also known as visceral fat grade, has proven to be a reliable indicator of visceral fat accumulation and dysfunction in adipose tissue (4). Krakauer (5) and others developed a comprehensive body measurement index integrating WC, BMI, and height, called the A Body Shape Index (ABSI), a recent research has shown that ABSI is positively correlated with abdominal fat tissue and significantly associated with mortality rates. The Body Roundness Index (BRI) proposed by Thomas et al. in 2013 (6) is a body measurement index calculated using waist circumference and height, which can assess the content of body fat, especially visceral fat; a higher BRI value indicates a greater deposition of visceral fat. The Conicity Index (C-index) is an indicator of central obesity calculated using waist circumference, weight, and height (7). Research indicates that the C-index can estimate abdominal fat quantity. In comparison to WC and BMI, the C-index not only assesses fat quantity in obese individuals but also in lean individuals.

Currently, most studies on the relationship between anthropometric measurements and T2DM focus on exploring their association with the occurrence of T2DM (8, 9). There are few studies about the relationship between the anthropometric measurements and the complications of T2DM patients. Currently, many studies have also focused on the correlation between obesity and DKD, a recent result provide genetic evidence for a causal link between obesity and DKD in diabetes. As obesity prevalence rises, this finding predicts an increase in DKD prevalence unless intervention should occur, which gave the genetic evidence for a causal role of obesity in DKD (10). Recently, some researches show that VAI was associated with increased likelihood of the decline in renal function. Higher VAI score was associated with increased risk of CKD, independently of established risk factors (11, 12).

This study aims to analyze the relationship between novel anthropometric measurements and DKD among patients with T2DM and to compare their ability to predict the occurrence of DKD with three conventional anthropometric indices related to obesity, including BMI, waist to height ratio (WHtR), and WHR. Additionally, it will provide novel clinical evidence for the early prevention of DKD in T2DM patients.

## Research subjects and method

### Ethics statement

The study was approved by the Ethics Committee of Peking University International Hospital and was conducted in accordance with the ethics standards of institutional and national research committees and the 1964 Helsinki Declaration and its later amendments or comparable ethics standards. As the study involved the retrospective analysis of clinical data, the requirement for written informed consent was waived.

### Research subjects

The present study conducted a retrospective analysis of a total of 1817 patients diagnosed with T2DM who were admitted to the Department of Endocrinology at Peking University International Hospital from January 2017 to August 2021. The study population comprised 1053 males and 764 females. All participants in this study were diagnosed with diabetes according to the diagnostic criteria for diabetes established by the World Health Organization (WHO) in 1999 (13). The criteria include :(1) Experiencing typical symptoms of diabetes with random blood glucose levels ≥ 11.1mmol/L; (2) fasting blood glucose levels ≥ 7.0mmol/L; and (3) blood glucose levels ≥ 11.1mmol/L during oral glucose tolerance tests or after ingesting 75g of glucose for 2 hours. Patients who did not exhibit any symptoms of diabetes were required to undergo repeated testing on another day. The exclusion criteria for this study were as follows: (1) patients with type 1 diabetes, gestational diabetes, and other special types of diabetes; (2) patients with acute complications of diabetes; (3) patients with urinary tract infection, hematuria (including the menstrual period), and non-DKD or ESRD requiring dialysis (4) patients with hematological diseases and malignant tumors.

## Methods

### General conditions

A retrospective analysis was conducted on the data of 1817 patients, and their general characteristics including sex, age, smoking, drinking, height, weight, waist circumference (WC), hip circumference (HC), systolic blood pressure (SBP), diastolic blood pressure (DBP), diabetic duration, and complications of diabetes were recorded in detail.

### Laboratory biochemical indices

All participants were required to fast for more than 8 hours, and venous blood was drawn on the following morning. The biochemical indices that were measured included glycosylated hemoglobin (HbA1c), fasting blood glucose (FBG), postprandial blood glucose (PBG), triglyceride (TG), total cholesterol (TC), high-density lipoprotein cholesterol (HDL-C), low-density lipoprotein cholesterol (LDL-C), serum creatinine (sCr) and uric acid (UA). The participants were asked to provide urine samples for three consecutive mornings. The samples were used to measure urinary microalbuminuria, urinary creatinine, and urinary albuminuria to creatinine ratio (UACR) were calculated.

### Diagnosis of DKD

According to the Chinese Guidelines for the prevention and treatment of DKD (14), DKD can be diagnosed in patients with at least one of the following conditions when DM is identified as the cause of kidney damage and other causes of CKD are excluded: (1) UACR ≥ 30 mg/g in at least two out of three tests, after excluding any interfering factors; (2) eGFR < 60ml · min^-1^ · (1.73m^2^) ^-1^ for more than 3 months; or (3) renal biopsy consistent with the pathological changes of DKD.

### Anthropometric indicators

The formulas for calculating anthropometric indicators are as follows:

1. Body mass index(BMI)=weight(kg)/height²(m²)
2. Waist-to-height ratio (WHtR)=WC (cm)/height (cm)
3. Waist-to-hip ratio (WHR) =WC(cm)/HC (cm)
4. Lipid accumulation product (LAP) (male) =[WC (cm) -65] ×TG
  LAP (female)=[WC (cm)-58] ×TG
5. A body shape index (ABSI)= WC(m)/[BMI^2/3^×height(m)^1/2^]
6. VAI (male) =WC (cm)/(39.68 + 1.88×BMI) ×TG/1.03×1.31/HDL-C
  VAI (female)=WC (cm)/(36.58 + 1.89×BMI) ×TG/0.81×1.52/HDL-C
7. Abdominal volume index (AVI)=[WC^2^(cm)+0.7×(WC-HC)^2^(cm)]/1000
8. Body roundness index (BRI)=364.2-365.5×[1-π^-2^× WC^2^(m)×height^-2^(m)]^1/2^
9. Conicity index (C-index)=0.109-1×WC(m)×[weight(kg)/height(m)]^-1/2^
10. Clinica Universidad de Navanra-Body Adiposity Estimator (CUN-BAE)=-44.988 + 0.503 × age +10.689 × sex +3.172 × BMI-0.026 × BMI^2^+0.181×BMI×sex-0.02×BMI×age-0.005×BMI^2^×sex+0.00021×BMI^2^×age(female=1, male=0)

According the level of VAI, subjects were divided into tertiles groups: (1) low VAI group (n=606):0.01-1.59; (2) middle VAI group(n=605):1.60-2.95; (3) high VAI group(n=606):2.96-55.29.

### Statistical analysis

The statistical analysis was performed using the SPSS Version 22.0 software (IBM, Chicago, IL, USA). Normality analysis was performed using the Kolmogorov–Smirnova test. The variables that followed normal distribution were presented as mean ± standard deviation. The comparison among the three groups was done using one-way analysis of variance (ANOVA), while the comparison between the two groups was done using the least significant difference (LSD) method. The comparison of counting data was done using the χ2 test. Logistic regression models were used for univariate analysis of the factors. In univariate regression analysis, factors with statistical significance are included in multivariate regression analysis. All data are divided into a training set and a validation set in a 2:1 ratio and internally validated. Multivariate logistic regression analysis was used to calculate odds ratios (ORs) and corresponding 95% confidence intervals (95%CIs).

We used the mean statistic to fill in missing values and the receiver operating characteristic (ROC) curve to evaluate the predictive ability of the model. The ROC curves were plotted and the area under the ROC curve (AUC) was calculated. On AUC, we select the value with the highest accuracy as the cut-off. To evaluate the discriminatory ability of the nomogram, we computed the AUC with a 95% CI by using 500 bootstrap resamplings. All statistical tests were two-sided, and p<0.05 was considered statistically significant.

## Results

### General situation of subjects classified according to the third percentile of VAI

The results showed that there were statistical differences in sex, age, WC, BMI, WHtR, WHR, VAI, LAP, ABSI, C-Index, CUN-BAE, SBP, DBP, HbA1c, FBG, PBG, UACR, TG, and HDL-C levels among the study subjects at different VAI levels (P all < 0.05). The results showed that with the increase of VAI level, the proportion of DKD gradually increased, and there was a statistical difference (P <0.05). With the increase of VAI levels, there is an increasing trend in male, age, WC, BMI, WHtR, WHR, VAI, LAP, ABSI, C-Index, CUN-BAE, SBP, DBP, HbA1c, FBG, PBG, UACR, TG, while HDL-C levels show a decreasing trend (P all <0.05) (**Table 1**).

**Table 1:** Comparison of general conditions and biochemical indexes among three groups.

Index	VAI			F(χ^2^)	p
	Low level (n=606)	Middle level (n=605)	High level (n=606)		
Age (years)	63.11 ± 8.69	63.28 ± 8.90 ^a^	63.61 ± 8.54^a^	8.08	<0.05
Sex (male%)	271 (44.72%)	342 (56.53%)	440 (72.61%)	307.68	<0.05
BMI (kg/m^2^)	23.95 ± 3.24	25.17 ± 3.29 ^a^	25.65 ± 3.33^a^ ^,^ ^b^	135.68	<0.05
Diabetic duration (years)	9.16 ± 7.29	9.30 ± 7.11	9.23 ± 6.84	1.49	0.21
SBP (mmHg)	122.60 ± 18.68	132.10 ± 17.96 ^a^	132.02 ± 15.55 ^a^	4.54	<0.05
DBP (mmHg)	78.93 ± 11.07	89.05 ± 11.35 ^a^	89.70 ± 10.82 ^a^	5.43	<0.05
Smoking	167 (27.56%)	196 (32.40%)	187 (30.86%)	11.15	<0.05
Drinking	81 (13.37%)	100 (16.53%)	104 (17.16%)	13.63	<0.05
WC (cm)	85.51 ± 10.61	89.98 ± 10.41 ^a^	91.94 ± 9.97 ^a^ ^,^ ^b^	197.57	<0.05
HC (cm)	95.80 ± 60.36	97.45 ± 45.34	97.88 ± 36.95	1.04	0.35
BRI	3.79 ± 1.30	4.38 ± 1.33 ^a^	4.79 ± 1.37 ^a^ ^,^ ^b^	278.99	<0.05
WHR	0.52 ± 0.07	0.55 ± 0.06 ^a^	0.57 ± 0.06 ^a^ ^,^ ^b^	36.88	<0.05
WHtR	0.93 ± 0.09	0.95 ± 0.08 ^a^	0.95 ± 0.07 ^a^ ^,^ ^b^	293.80	<0.05
VAI	1.05 ± 0.33	2.19 ± 0.38 ^a^	5.54 ± 3.55 ^a^ ^,^ ^b^	2449.45	<0.05
LAP	22.11 ± 13.30	42.91 ± 17.92 ^a^	92.22 ± 54.36 ^a^ ^,^ ^b^	2179.89	<0.05
ABSI	0.07 ± 0.01	0.08 ± 0.01 ^a^	0.09 ± 0.01 ^a^ ^,^ ^b^	74.26	<0.05
C-index	1.25 ± 0.12	1.29 ± 0.12 ^a^	1.31 ± 0.11 ^a^ ^,^ ^b^	136.39	<0.05
CUN-BAE	29.23 ± 6.95	32.11 ± 7.04 ^a^	34.54 ± 7.12^a^ ^,^ ^b^	277.06	<0.05
AVI	9.98 ± 35.51	9.64 ± 25.34	9.51 ± 20.29	0.13	0.88
TC (mmol/L)	4.71 ± 12.78	4.66 ± 1.78	5.05 ± 10.73	0.91	0.41
TG (mmol/L)	0.97 ± 0.34	1.57 ± 0.44 ^a^	3.02 ± 1.54 ^a^ ^,^ ^b^	2408.93	<0.05
LDL-C (mmol/L)	2.84 ± 8.98	2.84 ± 1.02	3.18 ± 10.80	1.13	0.32
HDL-C (mmol/L)	1.57 ± 4.18	1.14 ± 0.28 ^a^	0.98 ± 0.22 ^a^	31.18	<0.05
UA (umol/L)	344.16 ± 165.48	330.23 ± 138.12	353.77 ± 194.66	0.36	0.70
eGFR (ml/min/1.73m^2^)	122.26 ± 75.52	121.04 ± 35.34	118.11 ± 29.87	0.78	0.46
UACR (mg/g)	68.39 ± 95.72	78.51 ± 41.21	115.02 ± 50.31 ^a^	3.21	<0.05
HbA1c (%)	8.17 ± 2.13	8.30 ± 1.93	8.64 ± 1.96 ^a^ ^,^ ^b^	28.49	<0.05
FBG (mmol/L)	8.35 ± 3.16	8.70 ± 3.15 ^a^	9.22 ± 3.25 ^a^ ^,^ ^b^	35.23	<0.05
PBG (mmol/L)	12.01 ± 3.74	12.02 ± 4.26	12.26 ± 4.03 ^a^ ^,^ ^b^	13.54	<0.05
DKD (%)	98 (16.17%)	102 (16.86%)	127 (20.96%)	18.46	<0.05

a compared with the low VAI group, the difference was statistically significant (p<0.05).

b compared with the medium VAI group, the difference was statistically significant (p<0.05). BMI is for body mass index, SBP is systolic blood pressure, DBP is for diastolic blood pressure, FBG is for fasting blood glucose, PBG is for postprandial blood glucose, HbA1c is for glycosylated hemoglobin, eGFR is for glomerular filtration rates, UA is for uric acid, UACR is for urinary albuminuria creatinine ratio, TC is for total cholesterol, TG is for triglycerides, LDL-C is for low density lipoprotein cholesterol, HDL-C is for high density lipoprotein cholesterol, DKD is for diabetic kidney disease, WC is for waist circumference, HC is for hip circumference, BMI is for body mass index, WHR is for waist-to-hip ratio, WHtR is for waist-to-height ratio, VAI is for visceral adipose index, BRI is for body roundness index, C-index is for Conicity index, AVI is for abdominal volume index, ABSI is for a body shape index, CUN-BAE is for Clínica Universidad de Navarra-Body Adiposity Estimator, LAP is for lipid accumulation product.

### Logistic regression analysis of anthropometric indicators and risk of DKD

In Model 2 (after adjusting age, sex, diabetic duration, smoking, drinking, BP, blood glucose and blood lipids), logistic regression showed that high level of VAI was an independent risk factor for DKD (OR=1.38, 95% CI 1.18, 1.63). Also, the high level of LAP was an independent risk factor for DKD (OR=1.31, 95% CI 1.11, 1.56) and CUN-BAE was an independent risk factor for DKD (OR=1.76, 95% CI 1.32, 2.35) (**Table 2**).

**Table 2:** Logistic regression analysis of Anthropometric Indicators and DKD.

Index	Model 1			Model 2		
	Crude OR	95%CI	p	Adjust OR	95%CI	p
VAI						
Low level	1			1		
Middle level	1.07	0.90,1.27	0.44	1.06	0.89, 1.25	0.53
High level	1.31	1.11,1.54	<0.05	1.38	1.18, 1.63	<0.05
LAP						
Low level	1			1		
Middle level	0.97	0.82,1.15	0.71	0.99	0.83,1.18	0.94
High level	1.28	1.09,1.50	<0.05	1.31	1.11,1.56	<0.05
ABSI						
Low level	1			1		
Middle level	0.93	0.61,1.26	0.15	0.88	0.58,1.11	0.25
High level	0.94	0.63,1.23	0.23	0.95	0.55,1.47	0.24
AVI						
Low level	1			1		
Middle level	0.83	0.70,0.98	<0.05	0.75	0.63,0.89	<0.05
High level	0.89	0.75,1.04	0.15	0.93	0.62,1.27	0.32
BRI						
Low level	1			1		
Middle level	0.79	0.67,0.93	<0.05	0.74	0.63,0.89	<0.05
High level	0.89	0.76,1.05	0.17	0.86	0.72,1.02	0.07
C-index						
Low level	1			1		
Middle level	0.74	0.63,0.87	<0.05	0.69	0.58,0.82	<0.05
High level	1.03	0.62,1.36	0.15	1.02	0.53,1.54	0.17
CUN-BAE						
Low level	1			1		
Middle level	1.07	0.91,1.26	0.43	1.34	1.11,1.61	<0.05
High level	1.03	0.87,1.21	0.75	1.76	1.32,2.35	<0.05

Model 1 did not adjust for other factors; Model 2 adjusted for age, sex, diabetic duration, smoking, drinking, blood glucose, blood lipid and BP.

### Logistic regression analysis of VAI and risk of DKD in subgroup analysis

In subgroup analysis, for patients<60 years old, after adjusting age, sex, diabetic duration, smoking, drinking, BP, blood glucose and blood lipids, high level of VAI was an independent risk factor for DKD (OR=1.59, 95% CI 1.09, 2.31); In both female and male patients, after adjusting age, sex, diabetic duration, smoking, drinking, BP, blood glucose and blood lipids, high level of VAI was also an independent risk factor for DKD (OR=1.53, 95% CI 1.10, 2.14; OR=1.03, 95% CI 1.01,1.06) (**Table 3**).

**Table 3:** Logistic regression analysis of VAI and DKD in the subgroup.

Index	Model 1			Model 2		
	Crude OR	95%CI	P	Adjust OR	95%CI	P
<60 years						
Low level	1	1		1	1	
Middle level	1.32	0.99, 1.75	0.06	1.32	0.98, 1.78	0.07
High level	1.79	1.36, 2.35	<0.05	1.59	1.09, 2.31	<0.05
≥60 years						
Low level	1	1		1	1	
Middle level	0.98	0.79, 1.21	0.86	0.97	0.77, 1.21	0.76
High level	1.20	0.98, 1.48	0.08	1.03	0.77, 1.38	0.82
Male						
Low level	1	1		1	1	
Middle level	1.03	0.82, 1.30	0.77	1.15	0.86, 1.55	0.34
High level	1.43	1.15, 1.78	<0.05	1.03	1.01, 1.06	<0.05
Female						
Low level	1	1		1	1	
Middle level	1.13	0.87,1.48	0.37	1.10	0.83,1.45	0.50
High level	1.71	1.32,2.19	<0.05	1.53	1.10,2.14	<0.05

Model 1 did not adjust for other factors; Model 2 adjusted for age, sex, diabetic duration, smoking, drinking, blood glucose, blood lipids and BP.

### Univariate predictive model of DKD with anthropometric indicators

**Figure 1:**
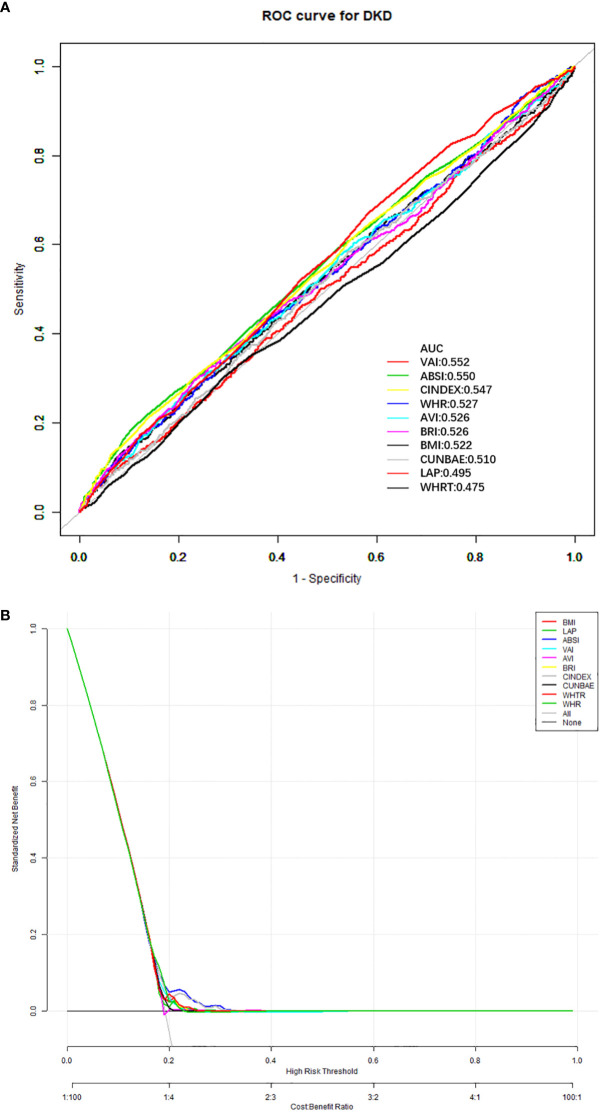
The ROC curve results of all anthropometric indicators for DKD prediction **(A)**, and decision curve analysis results of all anthropometric indicators for DKD prediction **(B)**. ROC, receiver operating characteristic; BMI, body mass index; WHR, waist-to-hip ratio; WHtR, waist-to-height ratio; VAI, visceral adipose index; BRI, body roundness index; C-index, Conicity index; AVI, abdominal volume index; ABSI, a body shape index; CUN-BAE, Clínica Universidad de Navarra-Body Adiposity Estimator; LAP, lipid accumulation product; DKD, diabetes kidney disease.

The model to predict the risk of DKD using anthropometric indicators, showed that the AUC of the models ranked VAI>ABSI>C-index>WHR>AVI=BRI>BMI>CUN-BAE>LAP>WHtR. The corresponding cut-off point of VAI for predicting DKD is 2.49 (**Table 4**, **Figure 1**).

**Table 4:** Univariate predictive model of DKD.

Index	AUC	95%CI	Cut-off point	Specificity	Sensitivity
BMI	0.53	0.51,0.55	26.85	0.75	0.31
WHTR	0.48	0.46,0.50	0.59	0.75	0.27
WHR	0.53	0.50,0.56	0.94	0.43	0.65
LAP	0.50	0.48,0.52	53.78	0.66	0.40
ABSI	0.55	0.53,0.57	0.08	0.82	0.27
VAI	0.55	0.53,0.57	2.49	0.60	0.49
AVI	0.53	0.49,0.53	7.07	0.72	0.32
BRI	0.53	0.50,0.54	3.49	0.73	0.32
C-index	0.55	0.52,0.58	1.23	0.74	0.33
CUN-BAE	0.51	0.48,0.52	33.74	0.42	0.60

BMI is for body mass index, WHR is for waist-to-hip ratio, WHtR is for waist-to-height ratio, VAI is for visceral adipose index, BRI is for body roundness index, C-index is for Conicity index, AVI is for abdominal volume index, ABSI is for a body shape index, CUN-BAE is for Clínica Universidad de Navarra-Body Adiposity Estimator, LAP is for lipid accumulation product.

### Multivariate predictive model for the risk of DKD with VAI

The multivariate predictive Model 1 was established with DKD as the dependent variable and sex, age, VAI, diabetic duration and HbA1c as independent variables. The regression equation is logit(DKD) = -3.46104 + 0.31954*(Sex=1) +0.00799*Age +0.04789*Duration +0.05080*VAI +0.07147*HbA1c.The AUC was 0.75 (95% CI 0.69, 0.80), the specificity was 76.75%, the sensitivity was 73.39%.

**Figure 2:**
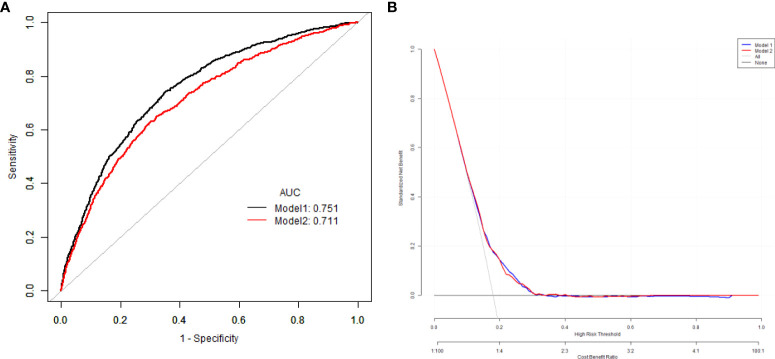
The comparison of the ROC curves **(A)**, and decision curve analysis results **(B)** of for the risk of DKD with VAI and BMI. ROC, receiver operating characteristic; BMI, body mass index; VAI, visceral adipose index; DKD, diabetes kidney disease.

The multivariate predictive Model 2 was established with DKD as the dependent variable and sex, age, BMI, diabetic duration, blood pressure, HbA1c as independent variables. The regression equation is logit(DKD) = -4.47680 + 0.25746*(Sex=1) +0.00724*Age +0.04833*Duration +0.04621*BMI +0.08197*HbA1c.The AUC was 0.71 (95% CI 0.68, 0.74), the specificity was 66.70%, the sensitivity was 71.65%. The predictive ability for DKD of Model 1 with VAI was higher than that of Model 2 with BMI (**Figure 2**).

## Discussion

At present, it is generally believed that T2DM is closely related to obesity. Insulin resistance(IR) and T2DM are considered as potential causes or contributing factors of many obesity-related diseases (15). In studies of fat distribution, the accumulation of adipose tissue in the upper body (abdominal region) has been associated with the development of obesity-related comorbidities and even all-cause mortality. Studies have shown that the accumulation of fat in the lower body (gluteofemoral region) is associated with protective lipids and reduces the prevalence of cardiovascular and metabolic diseases after adjusting for total body fat mass (16). There is a significant association between visceral fatty obesity in abdominal obesity and DM. There are two main types of adipose tissue in mammals, white adipose tissue (WAT) and brown adipose tissue (BAT), with WAT constituting a large proportion of the whole body and can be found in the abdominal cavity and around major organs and blood vessels under the skin. WAT stores excess energy in the form of TG, and increased accumulation of WAT, especially in visceral reservoirs, is a key determinant of cardiometabolic disorders, relative risk of hypertension. Studies have confirmed a significant association between visceral fatty obesity in abdominal obesity and diabetes (17). A large number of studies have shown that visceral fat can lead to elevated oxidative stress and chronic low-grade inflammation in the body, and high levels of inflammation-inducing factors such as IL-1β, IL-6 and TNF-α interfere with the normal metabolic signal of insulin (18). The main cause is adipose tissue abnormalities, such as adipocyte hypertrophy, abdominal obesity and ectopic fat deposition, which lead to systemic inflammation and metabolic dysfunction. The occurrence of microvascular complications in T2DM is directly proportional to fat content or body weight. The main reason is abnormal adipose tissue, such as adipocyte hypertrophy, abdominal obesity, and ectopic fat deposition, leading to systemic inflammation and metabolic dysfunction. At the molecular level, a research has demonstrated that macrophage restrictive protein tyrosine phosphatase 1B (PTP1B) is a key regulatory factor in metabolic syndrome inflammation involving insulin resistance, and PTP1B dysregulation may be the basis for retinal microvascular disease (19).

Allostatic load (AL) is a multisystem indicator of biological wear associated with adverse health outcomes (20). Allostatic load can lead to the occurrence of obesity, researchers have discovered that patients with end stage DKD often exhibit significant disruptions in their gut microbiota, immune imbalances, and allostatic loads, with the severity of gut microbiota imbalance being closely tied to the degree of renal injury (21). Increased carbonylation and glycation of proteins, one of the main manifestations of allostatic load (22), disrupts the structure and function of various proteins in DKD, leading to cell dysfunction and organ damage. Lower cumulative lifetime socioeconomic status was substantially associated with CKD prevalence but modestly with CKD incidence and eGFR decline via baseline allostatic load (23).

Complications of T2DM occur in direct proportion to fat content or body weight. The main cause is adipose tissue abnormalities such as fat cell hypertrophy, abdominal obesity and ectopic fat deposition, leading to systemic inflammation and metabolic dysfunction. Traditionally, obesity-related anthropometric measures are assessed, including WC, BMI, WHtR, WHR and so on. In recent years, some new anthropometric indicators reflecting abdominal obesity have been developed to evaluate the occurrence and development of obesity-related diseases such as diabetes, coronary heart disease and hypertension. At present, studies have confirmed that new anthropometric indicators can predict the occurrence of T2DM (24). However, there are few studies on the relationship between these novel anthropometric indicators and T2DM complications.

VAI is a sex-specific index of visceral obesity based on BMI, WC, TG and HDL-C. Yu et al. (25) conducted a cohort study in 7245 participants, showing that patients with a transition pattern of maintaining high VAI, high to low VAI, and low to high VAI had a higher risk of T2DM compared to those who maintained a low VAI pattern during follow-up. The result demonstrated a significant association of baseline VAI and its shifts with the risk of new-onset T2DM. Early prevention efforts are needed to control the development of T2DM in China with high VAI levels. In a health management cohort study in Beijing that followed 22,013 patients with T2DM, high level of VAI was significantly associated with increased risk of kidney disease (26). Hulkiti et al. (27) study found that high level of VAI was associated with an increased risk of microvascular complications such as retinopathy, nephropathy, and neuropathy, and could be used as a screening tool for T2DM patients. In a cross-sectional study in T2DM patients in Pakistan, VAI was found to be significantly positively associated with diabetes risk factors such as random blood glucose, uric acid (28). Similarly, in a cohort study in a Japanese population conducted by Shang et al. (29), VAI was found to be significantly associated with an increased risk of new onset T2DM in Japanese adults. In our study, the results showed that with the increase of VAI level, the proportion of DKD gradually increased, and there was a statistical difference. After adjusting age, sex, diabetic duration, smoking, drinking, BP, blood glucose and blood lipids, the high level of VAI was an independent risk factor for DKD (OR=1.38, 95% CI 1.18, 1.63).In subgroup analysis, for patients<60 years old, high level of VAI was an independent risk factor for DKD (OR=1.59, 95% CI 1.09, 2.31); in both female and male patients, high level of VAI was also an independent risk factor for DKD (OR=1.53, 95% CI 1.10, 2.14; OR=1.03, 95% CI 1.01,1.06). Similar to our findings, in a study of 4658 patients with DM, the VAI level was significantly associated with a greater prevalence of cardiovascular disease (OR 1.35; 95% CI 1.13, 1.62) and DKD (OR 1.38; 95% CI 1.12, 1.70) (30). Another study similarly concluded that VAI was an independent risk factor for the development of DKD (OR=1.03) (31). However, these two studies did not carry out further analysis of the cut point of VAI for predicting DKD, and our study found that the cut-off points of VAI for predicting DKD is 2.49. The clinical application value is that when the VAI of patients T2DM exceeds cut off clinically, the possibility of DKD is very high, so it provides a basis for the early diagnosis and treatment of DKD. In order to avoid the progression to DKD, the level of VAI should be controlled below the cut off level. At the same time, among all anthropometric indicators, we found that VAI had the strongest predictive ability for DKD. Among the prediction models of DKD, the AUC of the prediction model containing VAI was higher than that of the prediction model containing BMI.

AVI is a anthropometric index calculated based on HC and WC proposed by Fernado (27) to estimate the total abdominal volume, including the volume of abdominal adipose tissue in theory. The cross-sectional studies conducted by AVI show that AVI is a reliable anthropometric tool, and the estimation of visceral fat by abdominal volume is closely related to T2DM. Studies have confirmed that AVI is an independent risk factor for T2DM and metabolic syndrome, and can be used to predict the occurrence of T2DM (24). Another recent study with a small sample found that AVI as a measure of general volume, can be used as surrogates in the evaluation of high cardiovascular risk among T2DM patients (32). In our study, our results showed that after adjusting for other factors, AVI was not an independent risk factor for DKD occurrence. AVI cannot accurately distinguish the difference in fat deposition between male and female individuals. Therefore, further confirmation is needed to determine whether it has predictive significance for visceral fat in clinical practice.

In 2012, Krakauer et al. (5) proposed A Body Shape Index (ABSI), which used BMI and height to adjust WC. The higher the value, the larger the WC under certain weight and height conditions, the more consistent with visceral obesity. Some studies have found that ABSI may be a visceral abdominal marker associated with adverse metabolic changes and can be used for risk assessment of atherosclerotic diseases in postmenopausal women (33). However, Japanese population studies have found that ABSI is no better predictor of the risk of DM, hypertension, and dyslipidemia than BMI or WC (34). A study of Caucasian Caucasians showed that the combination of BMI and ABSI provided a better assessment of metabolic risk factors than BMI or ABSI alone (35). A recent Mendelian randomization showed that the risk of T2DM increased for genetically high ABSI in women (36). In our study, our results showed that after adjusting for other factors, ABSI was not an independent risk factor for DKD occurrence. Given that people of different races have different formulas for calculating ABSI, the key to its application lies in selecting the appropriate formula for scientific research.

There are also some limitations in our study. First, since it was a retrospective study, prospective studies are required to explore the underlying mechanism and pathway of the relationship between VAI and DKD. Second, the subjects of this study were all inpatients, which may not fully represent the correlation between and DKD in outpatients with T2DM. Finally, maybe due to the limitation of the study sample size, the ROC results need to be further improved. Nevertheless, the current research on the relationship between VAI and DKD is limited, and our findings are only a preliminary exploration. Due to the lack of externally validated population data, the predictive ability of the model is limited. We will supplement this limitation in future research and have included it in the revised manuscript. In addition, our study was a retrospective study conducted among hospitalized patients, which may have limitations. In future studies, we will further expand the sample size and conduct multicenter studies to improve the research results of this study.

## Conclusion

In conclusion, this study found that patients with high level of VAI had a significantly higher incidence of DKD. After adjusting age, sex, diabetic duration, smoking, drinking, BP, blood glucose and blood lipids, high level of VAI was an independent risk factor for DKD and the cut-off point of VAI for predicting DKD is 2.49. These findings may be useful in predicting DKD in patients with T2DM.

## Data Availability

The raw data supporting the conclusions of this article will be made available by the authors, without undue reservation.
